# Co-delivery of nanoparticle and molecular drug by hollow mesoporous organosilica for tumor-activated and photothermal-augmented chemotherapy of breast cancer

**DOI:** 10.1186/s12951-021-01025-w

**Published:** 2021-09-27

**Authors:** Haixian Zhang, Feifei Song, Caihong Dong, Luodan Yu, Cai Chang, Yu Chen

**Affiliations:** 1 Department of Ultrasound, Fudan University Shanghai Cancer Center; Department of Oncology, Shanghai Medical College, Fudan University, 200032 Shanghai, People’s Republic of China; 2 Department of Pathology, Shanghai Tenth People’s Hospital Affiliated to Tongji University, 200072 Shanghai, People’s Republic of China; 3 Department of Ultrasound, Zhongshan Hospital, Fudan University and Shanghai Institute of Medical Imaging, Shanghai, 200032 People’s Republic of China; 4 Materdicine Lab, School of Life Sciences, Shanghai University, Shanghai, 200444 People’s Republic of China

**Keywords:** Disulfiram, Mesoporous organosilica, Copper, Photothermal, Breast cancer

## Abstract

**Background:**

In comparison with traditional therapeutics, it is highly preferable to develop a combinatorial therapeutic modality for nanomedicine and photothermal hyperthermia to achieve safe, efficient, and localized delivery of chemotherapeutic drugs into tumor tissues and exert tumor-activated nanotherapy. Biocompatible organic–inorganic hybrid hollow mesoporous organosilica nanoparticles (HMONs) have shown high performance in molecular imaging and drug delivery as compared to other inorganic nanosystems. Disulfiram (DSF), an alcohol-abuse drug, can act as a chemotherapeutic agent according to its recently reported effectiveness for cancer chemotherapy, whose activity strongly depends on copper ions.

**Results:**

In this work, a therapeutic construction with high biosafety and efficiency was proposed and developed for synergistic tumor-activated and photothermal-augmented chemotherapy in breast tumor eradication both in vitro and in vivo. The proposed strategy is based on the employment of HMONs to integrate ultrasmall photothermal CuS particles onto the surface of the organosilica and the molecular drug DSF inside the mesopores and hollow interior. The ultrasmall CuS acted as both photothermal agent under near-infrared (NIR) irradiation for photonic tumor hyperthermia and Cu^2+^ self-supplier in an acidic tumor microenvironment to activate the nontoxic DSF drug into a highly toxic diethyldithiocarbamate (DTC)-copper complex for enhanced DSF chemotherapy, which effectively achieved a remarkable synergistic *in-situ* anticancer outcome with minimal side effects.

**Conclusion:**

This work provides a representative paradigm on the engineering of combinatorial therapeutic nanomedicine with both exogenous response for photonic tumor ablation and endogenous tumor microenvironment-responsive in-situ toxicity activation of a molecular drug (DSF) for augmented tumor chemotherapy.

**Graphical abstract:**

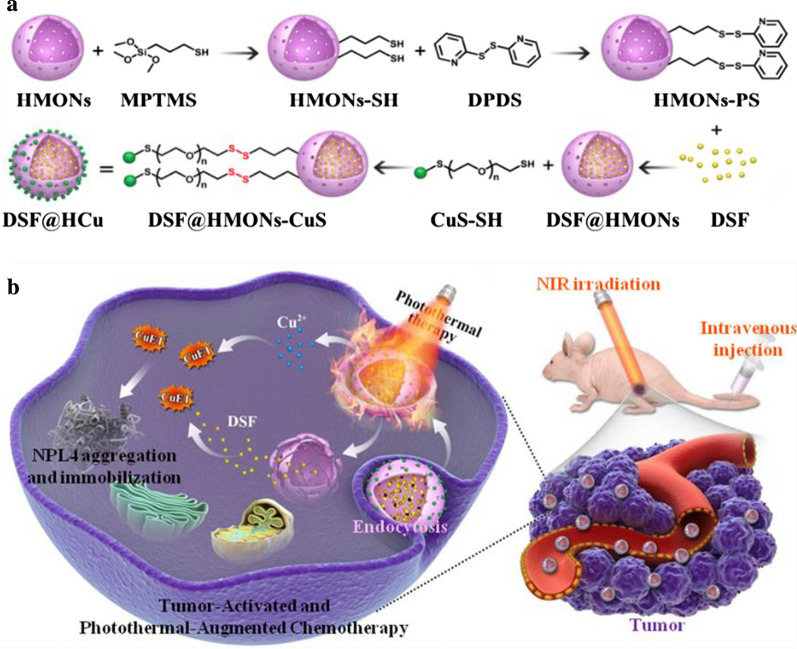

**Supplementary Information:**

The online version contains supplementary material available at 10.1186/s12951-021-01025-w.

## Background

Drug delivery nanosystems have been employed for efficient cancer diagnosis and treatment, particularly since the significant development of nanobiotechnology applications [1–8]. These organic and inorganic nanoplatforms are multifunctional, have excellent biocompatibility, have relatively high stability in bodily fluids, and have the capacity for the controlled release of therapeutic agents from the nanocarriers in the desired sites, particularly toward tumor cells [9–18]. In particular, biocompatible organic–inorganic hybrid hollow mesoporous organosilica nanoparticles (HMONs) have shown high performance in molecular imaging and drug delivery as compared with other inorganic nanosystems [19–24]. Benefited by the enhanced permeability and retention (EPR) effect [25–28], the nanosystems could deliver relatively more drugs to the desired sites. However, most drugs still accumulated in major organs, such as the liver and spleen, causing undesirable side effects [29–31]. Herein, we adopted the molecular drug disulfiram (DSF), a United States Food and Drug Administration (US FDA)-approved alcohol-abuse drug, as a chemotherapeutic agent given its recently reported effectiveness for cancer chemotherapy, especially for breast cancer [32–34]. It has been revealed that the anticancer toxicity of DSF was copper-dependent. As such, DSF-based chemotherapy is largely dependent on the number of Cu^2+^ ions present at the tumor site, which can maximize the chemotherapeutic efficacy of DSF [32, 33, 35–39].

**Fig. 1:**
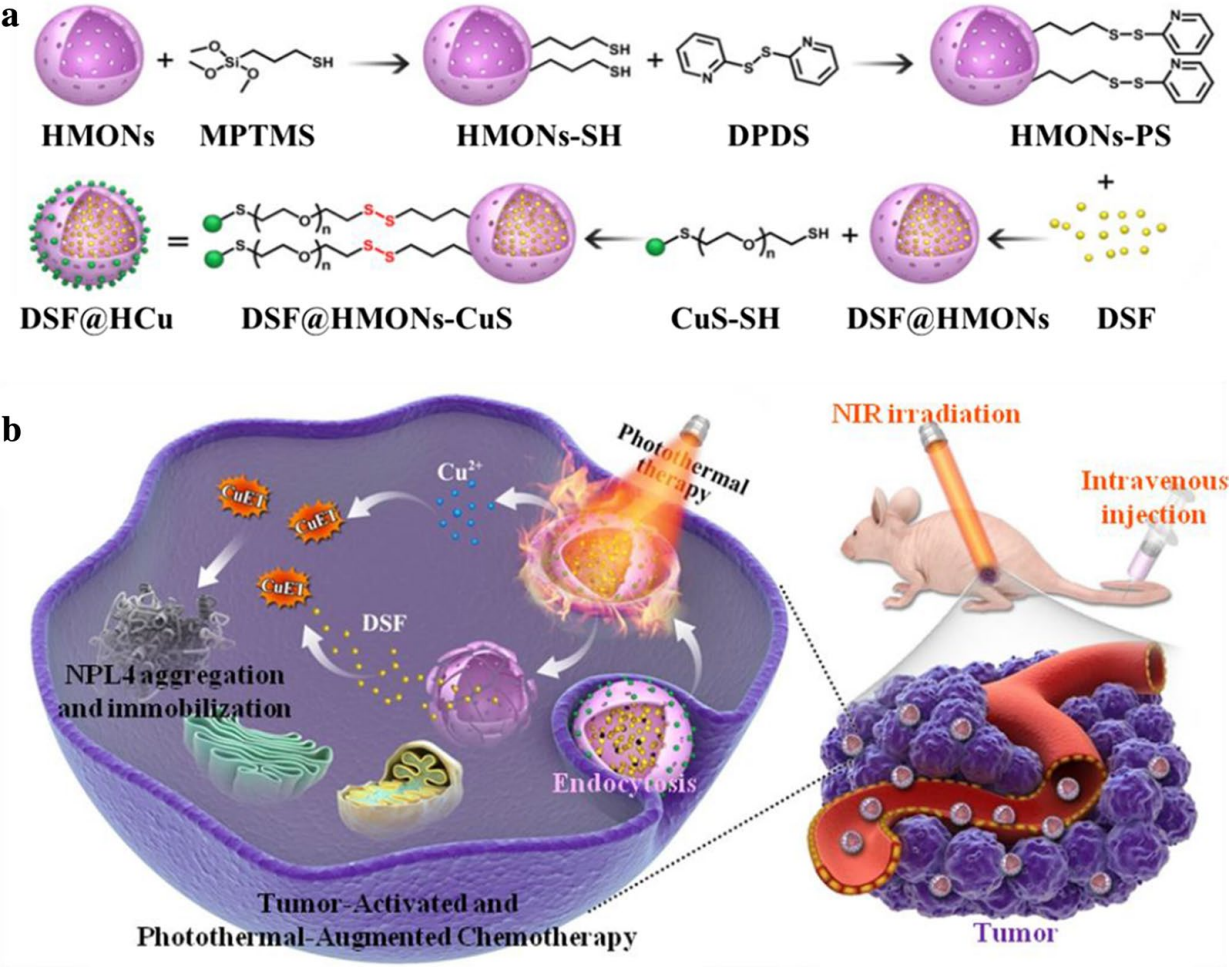
**a** Schematic illustration of the stepwise construction of DSF@HCu nanomedicine, and (**b**) NIR photothermal-augmented and *in-situ* Cu^2+^ release-activated DSF chemotherapy for the efficient killing of cancer cells and suppression of tumor growth with minimized side effects

In addition, the breast tumor is a superficial tissue mass, to which photothermal therapy (PTT) can be explored for tumor ablation [6, 11, 40–44]. Near-infrared (NIR) light is a low energetic (safe) light that can effectively penetrate the breast tissues. Herein, ultrasmall CuS nanoparticles, which served as Cu^2+^ self-providers and photothermal agents, were decorated onto the surface of HMONs via a disulfide linker (HMONs-ss-CuS, designated as HCu), while the chemotherapeutic drug DSF was encapsulated into the mesopores and hollow interior of HMONs, finally designated as DSF@HCu (Fig. 1a). As such, the tail-vein injection of the engineered DSF@HCu multifunctional nanomedicine was implemented into the bloodstream, which allowed for its efficient accumulation in the tumor tissues due to the typical EPR effect. Hence, the concurrent introduction of DSF@HCu theranostic nanosystems and the accompanying photothermal hyperthermia were rationally designed to achieve the enhanced and synergistic therapeutic outcome with exogenous photonic irradiation and endogenous tumor microenvironment (TME)-responsive toxicity activation of the chemotherapeutic drug with minimized side effects against breast cancer (Fig. 1b).

## Results

### Synthesis and characterization of HCu nanosystems

**Fig. 2:**
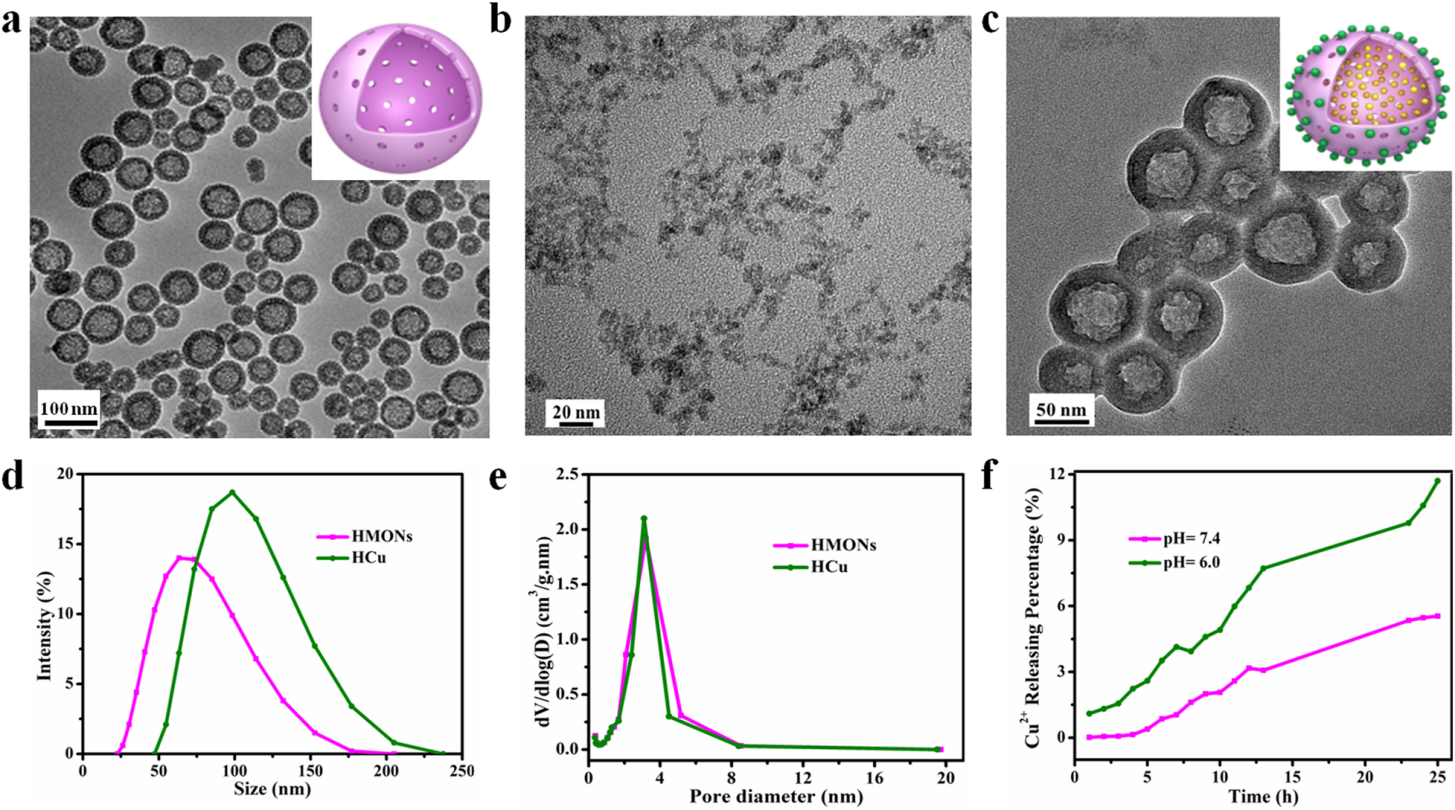
Morphology and structure characterization of HCu. TEM images of (**a**) HMONs, **b** CuS, and **c** HCu. **d** The average particle size of HMONs and HCu was determined by DLS. **e** The corresponding pore-size distributions of HMONs and HCu. **f** The releasing profiles of Cu in the buffer solutions with varied pH conditions (pH = 6.0 and pH = 7.4)

HMONs were synthesized using SiO_2_ as the hard template based on our previously developed “structural difference-based selective etching” method. In addition, bis(3-triethoxysilylproyl)disulfide (BTES) was employed as the organosilica shell with a disulfide bond-incorporated framework, which was followed by etching SiO_2_ core away under an alkaline condition [45, 46]. The transmission electron microscope (TEM) image results (Fig. 2a) indicated that the HMONs had a highly dispersive and uniform spherical morphology as well as a hollow structure. Initially, sulfhydryl groups were added to the as-synthesized HMONs via typical 3-mercaptopropyltrimethoxysilane (MPTMS) grafting (HMONs-SH), thereby obtaining multifunctional theranostic nanosystems. Subsequently, the anticancer drug DSF was encapsulated into the hollow interior of HMONs-SH via physical adsorption (DSF@HMONs-SH). The DSF encapsulation efficiency was calculated to be 20.9%. The DSF@HMONs-SH surface was then covalently conjugated with ultrasmall CuS-PEG-SH nanoparticles (Fig. 2b) via disulfide linkers (designated as DSF@HCu, Fig. 2c). The average size of the HMONs was approximately 73.4 nm as tested by dynamic light scattering (DLS), which increased to 98.4 nm due to CuS conjugation (Fig. 2d). Moreover, the initial mesopore size of the HMONs was 3.2 nm, which exhibited negligible mesopore size change (3.1 nm) after CuS-PEG-SH conjugation, indicating that the mesoporous channel was not blocked after conjugation with the CuS-PEG-SH nanoparticles on the surface of the HMONs (Fig. 2e). The Cu^2+^-releasing pattern revealed enhanced Cu^2+^ release under acidic conditions. Only 5.54% of Cu^2+^ was released after 24 h in a neutral environment (pH = 7.4), and elevated to 11.7% in an acidic environment (pH = 6.0) (Fig. 2f). In addition, the DSF-releasing pattern, as shown in Additional file 1: Figure S1, further reveals that DSF encapsulated in the hollow interior and mesopores was highly stable in aqueous buffer, where the releasing amount of DSF after 24 h was only 1.18% in aqueous solution. These data illustrate the enhanced Cu^2+^ self-supply from HCu in the acidic environment, which accompanied the release of DSF that was encapsulated in the hollow interior.

**Fig. 3:**
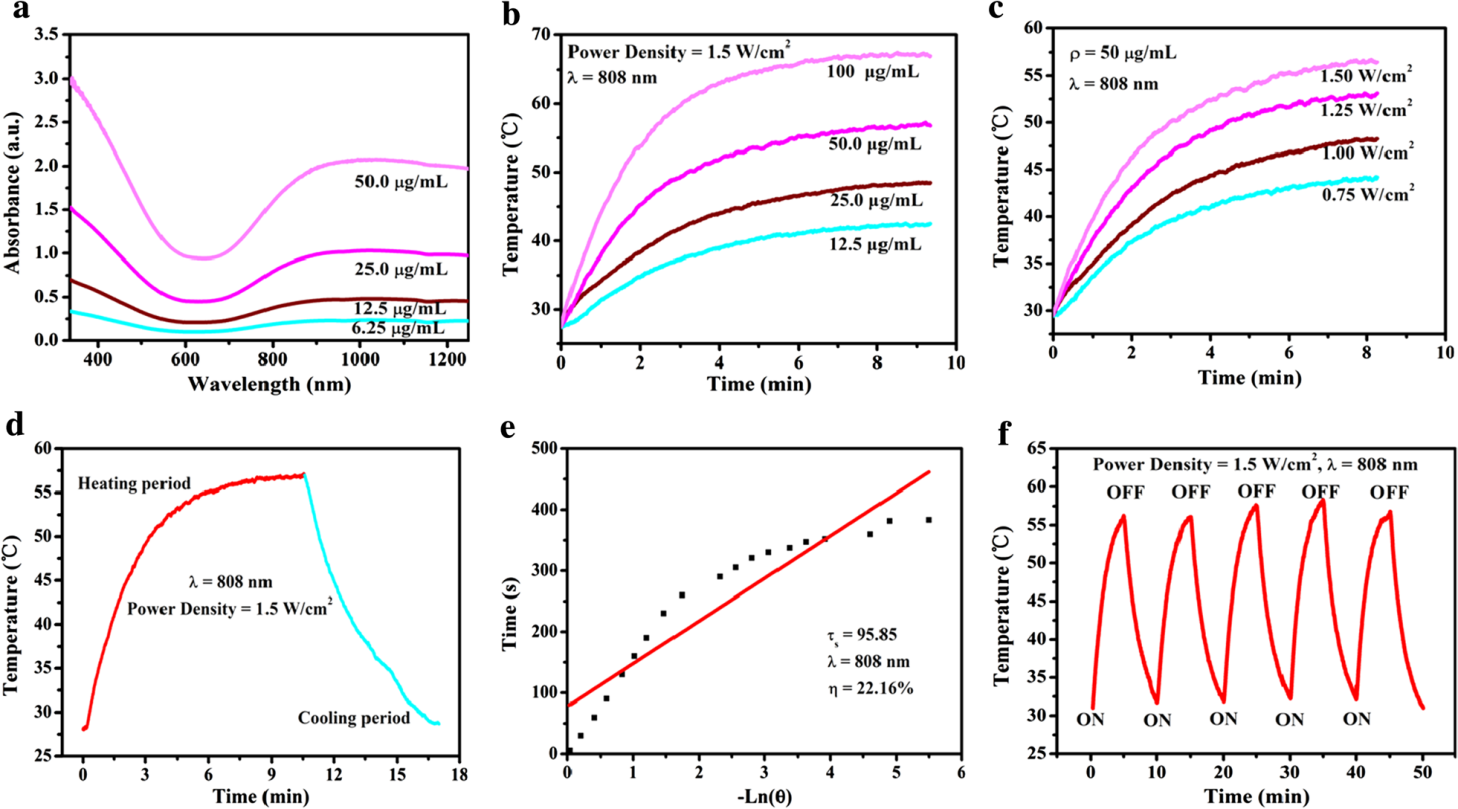
In vitro photothermal-conversion assessment of HCu. **a** UV–vis-NIR spectra of HCu at different concentrations (6.25, 12.5, 25, and 50 μg/mL) in aqueous solution. **b** Temperature changes of HCu aqueous solution with NIR laser (808 nm, power density: 1.5 W/cm^2^) irradiation at elevated concentrations (12.5, 25, 50, and 100 μg/mL). **c** Photothermal-heating curves of HCu dispersed in the aqueous solution irradiated by different power intensities (0.75, 1.0, 1.25, and 1.5 W/cm^2^) of NIR laser at the wavelength of 808 nm. **d** Photothermal performance of HCu dispersed in aqueous solution under NIR irradiation; the laser was turned off when the temperature was stable. **e** Time constant for heat transfer calculated from the cooling period. **f** Heating curve of HCu dispersed in water for five laser on/off cycles irradiated by 808 nm laser at the power intensity of 1.5 W/cm^2^

The therapeutic HCu nanosystem maintained the high photothermal-conversion capability of CuS. The ultraviolet–visible-infrared (UV–vis-NIR) spectra of CuS and HCu exhibited strong photo-absorption in the NIR range (Additional file 1: Fig. S2a, Fig. 3a). The photothermal-conversion performance of CuS and HCu was investigated at different concentrations (12.5, 25, 50, and 100 μg/mL) by exposing the aqueous solutions to 808 nm NIR laser at a power density of 1.5 W/cm^2^ (Additional file 1: Fig. S2b, Fig. 3b). The temperature reached as high as 54.3 °C when the concentration of HCu was set at 50 μg/mL, which is high enough to kill cancer cells. The temperature curve of CuS and HCu at various power densities of the 808 nm laser (0.75, 1.0, 1.25, and 1.5 W/cm^2^) indicates that the thermal effect is power density-dependent (Additional file 1: Fig. S2c, Fig. 3c). The laser was turned off when the temperature was stable, according to the previous report on the calculation of the photothermal-conversion efficiency [47–49], and the photothermal-conversion efficiency (η) of HCu was calculated to be 22.16% (Additional file 1: Fig. S2d, e, Fig. 3d, e). The heating curve showed that the thermal effect of CuS and HCu had not visibly changed during five laser on–off heating cycles (Additional file 1: Fig. S2f, Fig. 3f), suggesting that both compounds had relatively high photothermal stabilities.

### In vitro photothermal ablation and Cu^2+^-activated DSF chemotherapy against 4T1 breast cancer cells

**Fig. 4:**
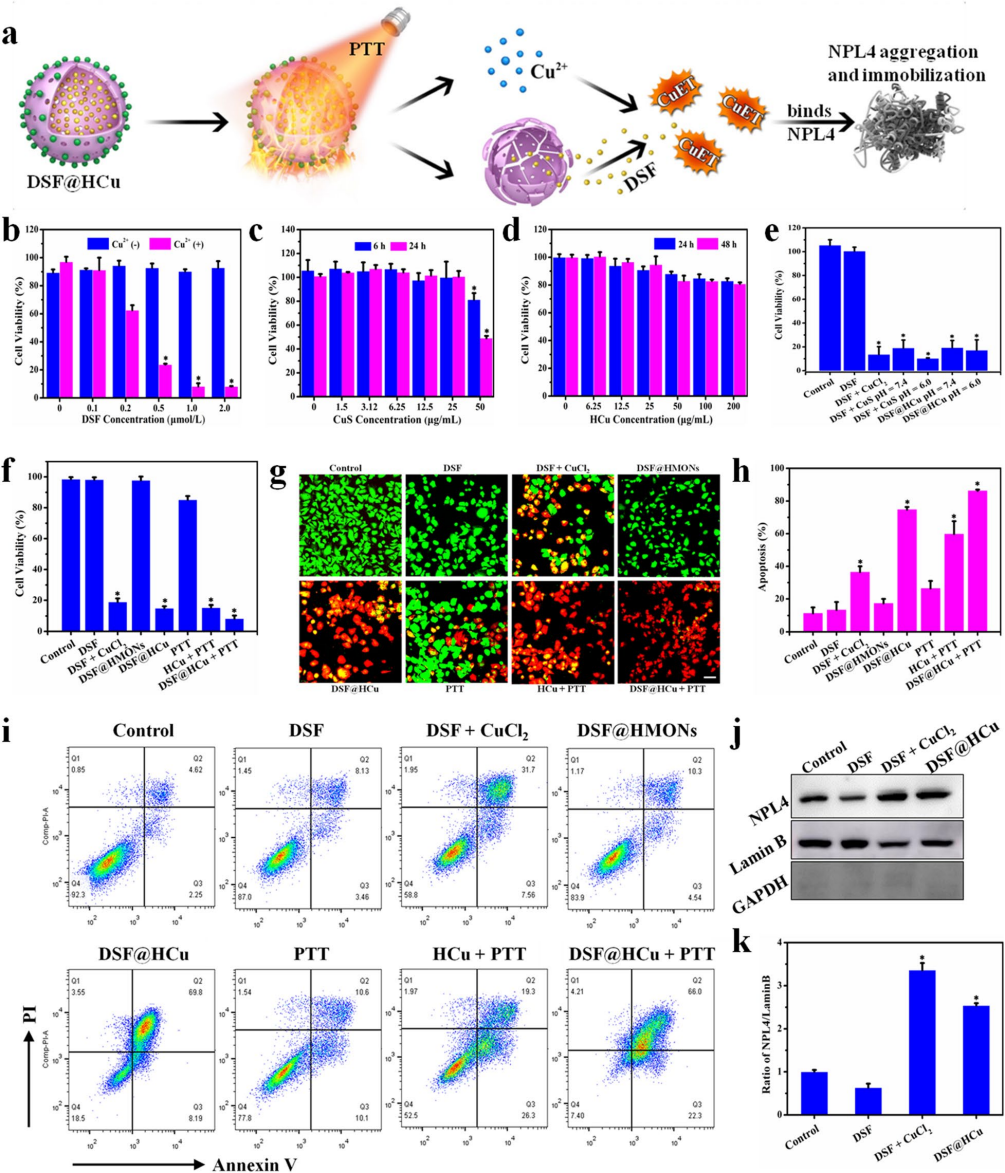
In vitro synergistic chemo-photothermal anti-cancer therapy. **a** Schematic illustration of the underlying mechanism of photothermal-augmented and DSF-based chemotherapy against breast cancer. **b** Relative cell viability of 4T1 cancer cells after incubation with DSF at elevated concentrations (0, 0.1, 0.2, 0.5, 1.0, and 2.0 μmol/L) with or without the addition of Cu^2+^. **c** Relative cell viability of 4T1 cancer cells after incubation with CuS at elevated concentrations (0, 1.5, 3.12, 6.25, 12.5, 25, and 50 μg/mL) for 6 and 24 h. **d** Relative cell viability of 4T1 cancer cells after incubation with HCu at elevated concentrations (0, 6.25, 12.5, 25, 50, 100, and 200 μg/mL) for 24 and 48 h. **e** Relative cell viability of 4T1 cells after different treatments, including control, DSF, DSF + CuCl_2_, DSF + CuS pH = 7.4, DSF + CuS pH = 6.0, DSF@HCu pH = 7.4, and DSF@HCu pH = 6.0. **f** Relative cell viability of 4T1 cancer cells after different treatments, including control, DSF, DSF + CuCl_2_, DSF@HMONs, DSF@HCu, PTT, HCu + PTT, and DSF@HCu + PTT. **g** CLSM images of 4T1 cancer cells after different treatments, as stained by calcein-AM (green) and PI (red). Scale bar: 40 μm. **h** Flow-cytometry apoptosis assay of 4T1 cancer cells after different treatments followed by staining with Annexin V-FITC/PI. **i** Representative FCM diagrams of 4T1 cells after different treatments. **j** Western blot shows CuET binds to and immobilizes NPL4. **k** Ratio of NPL4/Lamin B. Error bars in the graphs (**b**–**f**, **h**) represent standard deviation (n = 5). Error bars in graph k represent standard deviation (n = 3). *p < 0.05, calculated by one-way ANOVA test

We initially systematically evaluated in vitro photothermal ablation-enhanced and Cu-activated DSF chemotherapy on the 4T1 breast cancer cells (Fig. 4a). First, because HMONs were used as the main component of the nanosystem, the cytotoxicity of the HMONs was initially evaluated. The HMONs did not reveal any obvious cytotoxicity against the 4T1 cancer cells even at a high concentration of 200 μg/mL after 48 h, indicating their biosecurity (Additional file 1: Fig. S3). To confirm the copper-dependent DSF anti-cancer bioactivity, the cytotoxicity of DSF at different concentrations (0, 0.1, 0.2, 0.5, 1.0, and 2.0 μmol/mL) with or without Cu^2+^ was investigated. The cell viability of DSF (0.2, 0.5, 1.0, and 2.0 μmol/mL) with Cu^2+^ dropped to 62.3%, 23.8%, 8.2%, and 8.2%, respectively, as compared with DSF of the same concentration without Cu^2+^ (94.3%, 92.7%, 90.2%, and 92.8%, respectively), indicating that DSF is an efficient copper-potentiated anti-cancer molecular drug (Fig. 4b). Based on the fact that CuS nanoparticles were employed as the Cu^2+^ donor, the cytotoxicity of CuS was then tested. The CuS nanoparticles themselves revealed no significant cytotoxicity against the 4T1 cancer cells at a concentration of 25 μg/mL in 24 h, demonstrating their desirable low cytotoxicity (Fig. 4c). The cytotoxicity of HCu at different concentrations (0, 6.25, 12.5, 25, 50, 100, and 200 μg/mL) was then investigated, to which the results revealed low cytotoxicity (200 μg/mL, 48 h) (Fig. 4d). Next, the 4T1 cancer cells were treated with PBS, DSF, DSF + CuCl_2_, DSF + CuS pH = 7.4, DSF + CuS pH = 6.0, DSF@HCu pH = 7.4, and DSF@HCu pH = 6.0. In the absence of CuS or HCu, the 4T1 cells maintained their viability (100%), whereas the addition of CuS or HCu resulted in a significant cell viability loss to 19.1% and 19.4%, respectively, especially under acidic conditions (10.3% and 17.2%, respectively). CuS and HCu substantially augmented the anti-cancer bioactivity of the DSF drug, especially in the acidic environment (Fig. 4e). Furthermore, to reveal the synergy of PTT for DSF-based chemotherapy, the 4T1 cancer cells were respectively treated with PBS, DSF, DSF + CuCl_2_, DSF@HMONs, DSF@HCu, PTT, HCu + PTT, and DSF@HCu + PTT. Without HCu, the 4T1 cells exhibited viability (85.2%) after being illuminated with an 808-nm laser at the power density of 1.5 W/cm^2^ for 10 min, whereas the addition of HCu or DSF@HCu resulted in a significantly lowered cell viability of 15.4% or 8.3%, respectively. The results demonstrated that the designed DSF@HCu nanomedicine could not only self-supply Cu^2+^ to significantly enhance the chemotherapeutic efficacy of DSF, but also acted as a photothermal agent to synergistically kill the breast cancer cells (Fig. 4f).

To directly observe and characterize the ratio of viable and dead cells after various treatments, calcein-AM/propidium iodide (PI) fluorescent dyes were used to label viable (green) and dead (red) 4T1 cancer cells. The results indicated that cell viability was not obviously affected in the control group as well as in the groups treated with DSF or DSF@HMONs. The PTT-treated group exhibited slight changes in their cell viability, as indicated by the strong green fluorescent signals of the viable cells. In comparison, DSF + CuCl_2_, DSF@HCu, HCu + PTT, and DSF@HCu + PTT killed a significant amount of 4T1 cancer cells, as presented by the strong red fluorescent signals. The DSF@HCu + PTT co-treatment terminated the majority of cancer cells (Fig. 4g). Furthermore, we also performed the apoptosis assay by Annexin V-FITC/PI staining and flow cytometry (FCM). The quantification results indicated that the apoptosis rate of the 4T1 cancer cells treated with DSF or DSF@HMONs was 13.4% or 17.3%, respectively, which was comparable with the control group (11.3%). The apoptosis rate was slightly upregulated after treatment with PTT alone (26.4%). However, the apoptosis rate of the 4T1 cells was dramatically increased after treatment with DSF + CuCl_2_, DSF@HCu, HCu + PTT, or DSF@HCu + PTT, which was 36.4%, 74.7%, 59.7%, or 86.2% respectively, of which the DSF@HCu + PTT group exhibited the maximum therapeutic efficacy (Fig. 4h, i).

It has been demonstrated that the efficient anti-cancer bioactivity of DSF was potentiated by copper, thus forming diethyldithiocarbamate (DTC)-copper complex (bis (diethyldithiocarbamate)-copper (CuET)) [50]. CuET binds to and immobilizes NPL4, which is an adaptor of p97 segregase, thereby allowing protein turnover for multiple regulatory and stress-response pathways in cells. Therefore, we extracted the nuclear protein of the 4T1 cells with different treatments and measured the immobilized NPL4 in the nucleus. The extracted nuclear protein was loaded for western blot with Lamin B, which only exists in the nucleus. GAPDH is located in the cytoplasm and is always set as loading control when measuring the target proteins that located in cytoplasm. Here, GAPDH was measured to make sure that the extracted nuclear protein was not mingled with cytoplasm protein. The observed accumulation by western blot verified the CuET-induced immobilization of endogenous NPL4. The results exhibited that the nuclear NPL4 protein of the 4T1 cancer cells was significantly increased after treatment with DSF + CuCl_2_ or DSF@HCu compared with the control group, which was 3.63- or 2.53-fold that of control, respectively (Fig. 4j, k). However, no accumulation of nuclear NPL4 was found in cells treated with DSF alone. It is reasonably deduced that the synergistic DSF@HCu-enabled chemotherapy and photothermal hyperthermia could also form CuET.

### In vivo biocompatibility and biodistribution evaluation

**Fig. 5:**
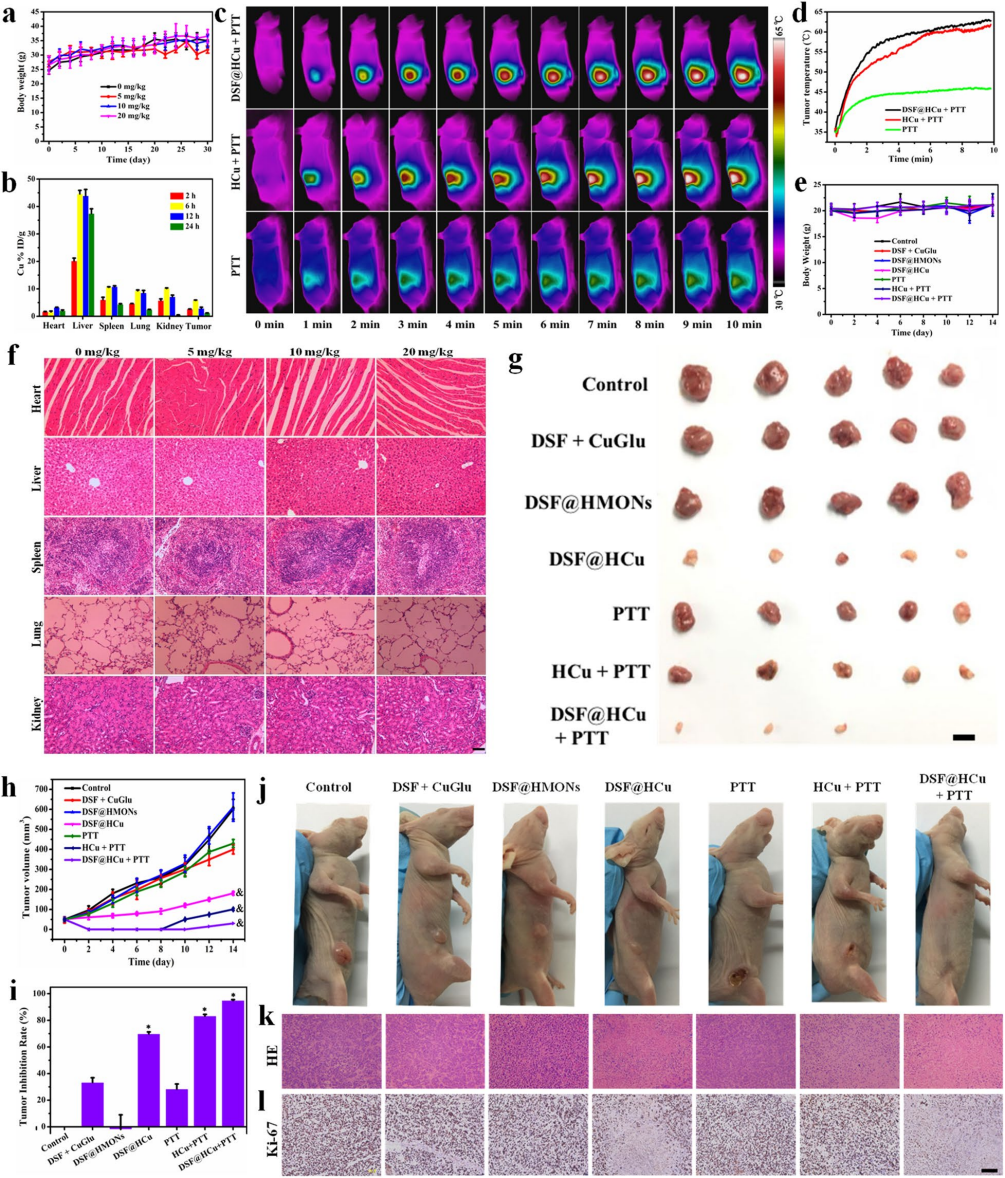
In vivo synergistic photothermal-augmented chemotherapy against 4T1 breast cancer. **a** Body-weight changes of the Kunming mice in the biocompatibility evaluation. **b** Body distribution of Cu in the major organs (heart, liver, spleen, lung, and kidney) and tumor after administration of HCu for varied time durations (2, 6, 12, and 24 h). **c** Temperature elevation of tumor region in 4T1 tumor-bearing mice under irradiation of 808-nm laser at the laser density of 1.5 W/cm^2^ for 600 s with or without the assistance of intravenously administrated HCu or DSF@HCu and (**d**) their corresponding heating curve. **e** Bodyweight changes of 4T1 tumor-bearing mice with different treatment: PBS (*i.v.*), DSF + CuGlu (*i.g.*), DSF@HMONs (*i.v.*), DSF@HCu (*i.v.*), PTT, HCu (*i.v.*) + PTT, and DSF@HCu (*i.v.*) + PTT. **f** H&E-stained histological images of the heart, liver, spleen, lung, and kidney in the biocompatibility evaluation. Scale bar: 50 μm. **g** Digital photographs of the dissected tumors from each group after 14 days of treatments. Scale bar: 1.0 cm. **h** Tumor volumes for each group of mice recorded every 2 days after different treatments. **i** Tumor inhibition rate from each group after 14 days of treatments. **j** Digital photographs of 4T1-tumorbearing mice and their tumor regions after different treatments. **k** H&E-stained histological images of tumor tissues. **l** Ki-67-stained cellular proliferation in tumor tissues sections. Scale bar: 50 μm. Error bars in the graphs (**a**, **b**, **e**, **h**, **i**) represent standard deviation (*n* = 5). *p < 0.05, calculated by one-way ANOVA test. ^&^p < 0.05, calculated by repeated measure ANOVA test

Prior to the subsequent therapeutic evaluation in vivo, a series of assessments were carried out to evaluate the in vivo biocompatibility and biodistribution of HCu. Healthy Kunming mice (*n* = 5 in each group) were administrated with HCu (0, 5 mg/kg, 10 mg/kg, and 20 mg/kg) through their tail veins. No evident major organ damage was observed within the one-month feeding term, as validated by the body weight (Fig. 5a), hematology (Additional file 1: Fig. S4a–d), serum biochemistry (Additional file 1: Fig. S4e, f) and histological analyses (Fig. 5f), thus indicating high biocompatibility of HCu to the animals within the tested dosage. The biodistribution of HCu in major organs and tumors signified that the HCu rapidly accumulated at the tumor within 6 h and attained the maximum tumor uptake of 5.8 ± 0.1% (Fig. 5b), guaranteeing synergistic therapeutics.

### In vivo synergistic anticancer efficacy of DSF@HCu nanomedicine by both exogenous photonic irradiation and endogenous TME activation

Further systematic in vivo anti-tumor assessments were conducted to accompany the observed intense NIR absorbance, high biocompatibility, high tumor accumulation, and in vitro high synergistic chemo-photothermal efficacy of DSF@HCu. The efficient accumulation of HCu or DSF@HCu into the tumor quickly elevated the tumor temperature upon 808 nm NIR laser irradiation (Fig. 5c). After intravenous administration of HCu or DSF@HCu, the tumor-site temperature of the 4T1 tumor-bearing mice increased from 35 °C to 61.8 °C or 62.8 °C, respectively, particularly after exposure to the 808 nm laser at a power density of 1.5 W/cm^2^ for 10 min (Fig. 5d), which was sufficiently high to eliminate the tumor tissue. In contrast, the tumor temperature in the saline-injected mice exhibited slight elevation (10 °C) under 808 nm NIR laser irradiation at the same corresponding laser power density. Then, the 4T1 tumor-bearing female BALB/c nude mice were randomly divided into seven groups, including saline (*i.v.*), DSF + CuGlu (*i.g.*), DSF@HMONs (*i.v.*), DSF@HCu (*i.v.*), PTT, HCu (*i.v.*) + PTT, and DSF@HCu (*i.v.*) + PTT. The body weight (Fig. 5e) and major organs (heart, liver, spleen, lung, and kidney) of the mice (Additional file 1: Fig. S5) did not exhibit any significant changes and damage throughout the therapeutic process, indicating negligible treatment side effects. More importantly, DSF@HCu exhibited high therapeutic efficacy and desirable biosafety.

The tumors in the HCu + PTT group were superficially and thermally eliminated at the early stage but reappeared after 8 days (Fig. 5h). Compared with the control group, the DSF@HCu group and HCu + PTT group exhibited tumor regression of 69.9% and 83.3%, respectively. Comparatively, with 808-nm laser irradiation, DSF@HCu presented effective growth suppression and the suppression rate was calculated to be 94.9% (Fig. 5i), suggesting that the synergetic photothermal-augmented chemotherapeutic efficacy was better than each aforementioned treatment alone.

According to the tumor-volume records (Fig. 5h) and corresponding digital pictures (Fig. 5g, j), the photothermal-augmented chemotherapy resulted in nearly complete tumor destruction without recurrence (tumor inhibition rate: 94.9%), demonstrating the potential of DSF@HCu nanomedicine as a powerful therapeutic agent for combating cancer. Hematoxylin–eosin (H&E) staining and Ki-67 immunohistochemistry staining (Fig. 5k, l) also respectively indicated significant histological damages and excellent tumor-cell inhibition in the tumor sections. The treatment group tumor cells shrank and shattered at different degrees, particularly following synergetic photothermal-augmented chemotherapy treatment, which was consistent with the above in vitro tests. These results indicated that the combination of DSF chemotherapy with PTT strengthened and enhanced the synergistic therapeutic capabilities compared with either individual DSF chemotherapy or PTT treatments.

## Discussion

We adopt the molecular drug DSF, a US FDA-approved alcohol-abuse drug, as a chemotherapeutic agent, which has been recently shown to be effective for cancer chemotherapy, especially for breast cancer. The ultrasmall CuS acted as both photothermal agent under NIR irradiation for photonic tumor hyperthermia and Cu^2+^ self-supplier in acidic tumor microenvironment to activate nontoxic DSF drug into highly toxic DTC-copper complex for enhanced DSF chemotherapy, which effectively achieved a remarkable synergistic *in-situ* anticancer outcome with minimal side effects. At cellular level, the mechanistic study uncovered that Cu^2+^-potentiated DSF nontoxic-toxic transformation, forming CuET, which binds to and immobilize NPL4, thereby allowing protein turnover for multiple regulatory and stress-response pathways in cells. This work provides a representative paradigm on the engineering of combinatorial therapeutic nanomedicine with both exogenous response for photonic tumor ablation and endogenous tumor microenvironment-responsive *in-situ* toxicity activation of the molecular drug for augmented tumor chemotherapy.

## Conclusions

In summary, this work proposed a distinct therapeutic modality of “endogenous tumor-activated and exogenous photothermal-augmented chemotherapy” based on the constructed DSF@HCu nanomedicine by the co-loading of CuS nanoparticles and molecular drug DSF into biocompatible and biodegradable HMONs. This therapeutic modality exhibited synergetic photothermal hyperthermia and *in-situ* tumor chemotherapy by copper self-supplied DSF toxicity activation. The efficient and synergistic therapeutic outcome was attributed to CuS-based photothermal-triggered cancer hyperthermia and Cu^2+^-potentiated DSF nontoxic-toxic transformation for cancer chemotherapy with tumor acidic microenvironment-triggered copper release. The systematic in vitro and in vivo evaluations demonstrated that the engineered DSF@HCu nanomedicine induced remarkable synergistic photothermal ablation and DSF chemotherapy under NIR light irradiation. These results demonstrated simultaneous high therapeutic outcome and mitigated side effects of the constructed DSF@HCu nanomedicine-photothermal hyperthermia with *in-situ* Cu^2+^-potentiated DSF chemotherapy for cancer-therapeutic modalities.

## Methods

### Materials

CuCl_2_·2H_2_O, sodium citrate, Na_2_S·9H_2_O, tetraethyl orthosilicate (TEOS), triethanolamine (TEA), hydrochloric acid (HCl, 37%), ethanol, and ammonia solution (25–28%) were purchased from Sinopharm Chemical Reagent Co. (Shanghai, China). Disulfiram (DSF), cetyltrimethylammonium chloride (CTAC, 25 wt%), 3-mercaptopropyltrimethoxysilane (MPTMS), and 2, 2′-dipyridyl disulfide (DPDS) were obtained from Sigma-Aldrich. Bis(3-triethoxysilylproyl)disulfide (BTES) was bought from Lark Chemical Technology Co., Ltd. HS-PEG-SH was purchased from Shanghai Yarebio Technology Co., Ltd. Calcein-AM/PI double staining kit was purchased from Beijing Solarbio Science & technology Co., Ltd. Cell counting kit-8 (CCK-8) was purchased from Dojindo Molecular Technologies. Annexin V-FITC/PI apoptosis detection kit was purchased from Vazyme (Nanjing, China). Cell culture medium 1640, trypsin–EDTA and fetal bovine serum (FBS) were provided by Gibco-BRL (Burlington, Canada). H&E and Ki-67 cell proliferation kit were purchased from Beyotime Biotechnology Co. (Haimen, China). The primary antibody rabbit anti-NPL4 antibody was bought from Abcam. The second antibody anti-rabbit IgG (horseradish peroxidase (HRP)-linked antibody) was bought from Cell Signaling Technology, Inc. All chemicals were used as received without further purification, and their aqueous solutions were prepared using deionized water.

### Preparation of HMONs

CTAC aqueous solution (2 g, 10 wt%) and TEA aqueous solution (0.08 g, 10 wt%) were mixed and magnetically stirred at 95 °C for 10 min. Then, TEOS (1 mL) was added into the above solution dropwisely. After 1 h, the mixture involving TEOS (1 mL) and BTES (0.6 mL) was added and the reaction was last for another 4 h. After centrifugation, the products were washed with ethanol for several times. Then, the template CTAC was removed by refluxing with a solution of HCl in ethanol (10% v/v) at 78 °C for 12 h. The extraction process was repeated for three times and the products were washed with deionized water for three times and stored in deionized water (20 mL). Finally, the sample (5 mL) was diluted in deionized water (100 mL), and then ammonia solution (2 mL) was added. The etching process was lasted for 3 h at 95 °C to obtain the final product HMONs.

### Synthesis of CuS and HS-PEGylated CuS nanoparticles

In a typical synthesis, 0.0855 g CuCl_2_·2H_2_O and 0.2279 g sodium citrate were dissolved in 100 mL distilled H_2_O under magnetic stirring for 30 min at room temperature, and then 1 mL 0.12009 g Na_2_S·9H_2_O aqueous solution was added to the mixture to immediately generate a black solution. After 5 min, the reaction mixture was heated to 90 °C and kept for 15 min before cooling down with ice, forming dark-green-colored citrate-coated CuS nanoparticles (Cit-CuS NPs). 0.2 g HS-PEG-SH was added to modify the surface of the Cit-CuS NPs at room temperature to obtained PEGylated CuS nanoparticles (CuS-PEG-SH NPs). The final product was stored at 4 °C for further characterization and use.

### Drug loading and preparation of DSF@HCu

First, HMONs (100 mg) was dispersed in isopropanol (100 mL), followed by adding MPTMS (200 μL) and refluxing at 80 °C overnight. After centrifugation and washing with methanol, the products (HMONs-SH) were dispersed in methanol (10 mL). DPDS (40 mg) was dispersed in methanol (5 mL), and then HMONs-SH (2 mL) was added and reacted at room temperature overnight to obtain the active HMONs-PS. HMONs-PS (10 mg) was dispersed in a DSF solution (2 mL chloroform, 40 mg/mL). DSF@HMONs was obtained after stirring at room temperature under dark condition for 24 h and collected by centrifugation. After centrifugation, the supernatant was collected and measured by UV–vis spectroscopy at λ = 262 nm to determine the DSF encapsulation efficiency. Encapsulation efficiency = (W_administered dose_–W_residual dose in solution_)/W_administered dose_ × 100%. Finally, DSF@HMONs-PS was reacted with CuS-PEG-SH to acquire the products DSF@HOMNs-CuS (designed as DSF@HCu).

### In vitro Cu^2+^ release at different pH values

To evaluate the Cu^2+^-releasing behavior, HCu (5 mg) was encapsulated into a dialysis bag (molecular weight cut-off = 5 kDa), and then immersed in PBS solution (25 mL) with different pH values (6.0 and 7.4). The release procedure was conducted in a shaking table (100 rpm, 37 °C) and the Cu^2+^-releasing amount was measured by an inductively-coupled plasma optical emission spectrometer (ICP-OES) at the given time points.

### Characterization

Transmission electron microscope (TEM) images were acquired on a JEOL 2011 TEM (JEOL Ltd., Tokyo, Japan) with an accelerating voltage of 200 kV. UV–vis spectra were recorded on a PerkinElmer Lambda 750 spectrophotometer. Dynamic light scattering (DLS) was performed on a Nano ZS90 Zetasizer (Malvern Panalytical, Malvern, UK) to determine the hydrodynamic particle size of the samples. Confocal laser-scanning microscope (CLSM) images were obtained on a FV1000 CLSM (Olympus, Tokyo, Japan). Quantitative elemental analysis was performed on an inductively-coupled plasma optical emission spectrometer (ICP-OES, Agilent Technologies, Santa Clara, CA, USA). Flow cytometry (FCM) analysis for apoptosis assays was performed using a BD LSRFortessa flow cytometer (Becton Dickinson, Franklin Lakes, NJ, USA). The western blot results were determined by Electro-Chemi-Luminescence display devices (Thermo Fisher Scientific, San Jose, CA, USA).

### Cell culture and animals

Mouse breast cancer cell line 4T1 cells were cultured in RPMI-1640 medium supplemented with 10% fetal bovine serum and 1% penicillin–streptomycin solution in a humidified incubator (5% CO_2_, 37 °C). Healthy BALB/c nude mice (18–22 g) and Kunming mice (24–28 g) were purchased from the Beijing Vital River Laboratory Animal Technology Co., Ltd. All animal experiments were performed under the guideline approved by the Animal Study Committee of Fudan University. For the breast cancer model, 4T1 cancer cells (5 × 10^5^) in PBS (5 μL) were implanted subcutaneously into the back of BALB/c nude mice.

### In vitro photothermal effect of HCu

The photothermal performance of HCu was characterized by recording the temperature changes during laser irradiation at 808 nm (FLIR TM A325SC camera). CuS and HCu were dispersed in deionized water with different Cu concentrations (12.5, 25.0, 50, and 100 μg/mL) and then exposed to 808 nm laser irradiation at the laser-power density of 1.5 W/cm^2^. In addition, the temperature increase of HCu aqueous solution at the Cu concentration of 50 μg/mL as irradiated by 808 nm laser at different power intensities (0.75, 1.0, 1.25, and 1.5 W/cm^2^) was tested.

### In vitro cytotoxicity assay

To evaluate in vitro cytotoxicity of CuS, a standard CCK-8 viability assay was conducted. Varied Cu concentrations of CuS (0, 1.5, 3.12, 6.25, 12.5, 25, and 50 μg/mL) were co-incubated with 4T1 cells pre-seeded in 96-well plates for 6 and 24 h. To evaluate the in vitro DSF anti-tumor activity enhanced by Cu^2+^, different DSF concentrations (0, 0.1, 0.2, 0.5, 1.0, and 2.0 μmol/L) with or without Cu^2+^ (10 μmol) were also co-incubated with 4T1 cells. Then CCK-8 diluted by RPMI-1640 at a ratio of 1:10 was added into plates to test the cell viabilities at a wavelength of 450 nm after 60 min on a microplate reader. To evaluate acid environment for Cu^2+^ release from CuS and HCu to enhance cytotoxicity of DSF, the wells were divided into seven groups, including control, DSF, DSF + CuCl_2_, DSF + CuS pH = 7.4, DSF + CuS pH = 6.0, DSF@HCu pH = 7.4, and DSF@HCu pH = 6.0.

### In vitro synergistic photothermal effect assisted by DSF@HCu

A standard CCK-8 protocol was used to evaluate the synergistic photothermal performance of DSF@HCu for killing cancer cells. 4T1 cells were seeded in 96-well plates in RPMI-1640 containing 10% FBS for 12 h to adhere to the plates at the density of 1 × 10^4^ per well. To evaluate the killing effect of synergistic therapy, the wells were divided into eight groups, including control, DSF, DSF + CuCl_2_, DSF@HCu, DSF@HMONs, PTT, HCu + PTT, and DSF@HCu + PTT. The laser-power density was set as 1.5 W/cm^2^. The Calcein-AM (15 μL) and PI (15 μL) dispersed in PBS (7.5 mL) were used to replace the cell culture media and stained the live (green) and dead (red) cells after varied treatments. After 15 min staining, the cells were washed by PBS for three times and observed by CLSM. Furthermore, the synergistic effect of chemo-photothermal therapy was confirmed by apoptosis assay and FCM measurement*.* Cells were plated in 6-well plates for 24 h before treatment. Then the cells were treated as described for indicated time and harvested for double staining with Annexin V-FITC/PI as manufacture’s instruction. The cells were then subjected to FCM measurement and analyzed with the FlowJo software system. The apoptosis rate was defined as the percentage of the Annexin V-FITC-positive cells.

### Anti-tumor mechanism of DSF@HCu

To investigate if DSF@HCu could also cause the same cascade reaction, CuET-induced immobilization of endogenous NPL4 accumulation was detected by nuclear protein extraction and western blot. The 4T1 cells were treated with control, DSF, DSF + CuCl_2_, or DSF@HCu for 24 h and collected for nuclear protein extraction according to the manufacturer’s instruction (Thermo scientific, cat# 78833). Briefly, the 4T1 cancer cells were trypsinized, washed with cold PBS, and lysed by ice-cold Cytoplasmic Extraction Reagent I (CER I) for 10 min. Ice-cold Cytoplasmic Extraction Reagent II (CER II) was added in it and then the mixture was vortexed vigorously twice and centrifuged at the maximum speed after incubation on ice for 5 min. The supernatant was collected as cytoplasmic protein and the pellet was suspended by Nuclear Extraction Reagent (NER) buffer, vortexed vigorously every 10 min for total four times and centrifuged at the maximum speed. The supernatant at the last step was the extracted nuclear protein. Equal amounts of nuclear protein were separated by SDS–PAGE and transferred to PVDF membranes (Bio-Rad). The membranes were blocked in 5% non-fat milk for 1 h at room temperature and then incubated with the primary antibodies diluted in blocking milk at 4 °C overnight. After washing three times with Tris-buffered saline containing 0.1% Tween, the membranes were incubated with HRP-conjugated secondary antibody diluted in blocking milk for 1 h at room temperature. Protein bands were detected with Enhanced Chemi-luminescence reagent (Millipore).

### In vivo biocompatibility evaluation

Healthy Kunming mice (n = 5 in each group) were administrated with HCu (0, 5 mg/kg, 10 mg/kg, and 20 mg/kg) through tail vein. Blood samples and major organs were collected from the control and treated groups at 30 d after injection. In addition, the body weight of mice was recorded every other day for 30 d. The heart, liver, spleen, kidney, and lung of different treatments were also harvested for H&E staining. H&E stained tissue sections were observed under an optical microscope.

### In vivo biodistribution evaluation

When the tumor volume of 4T1 breast cancer-bearing mice grew to approximately 100 mm^3^, a HCu saline solution (100 μL, 20 mg/kg) was injected into mice (n = 5) via the tail vein. Next, 2, 6, 12, or 24 h after the injection, mice were sacrificed and their major organs (heart, liver, lung, spleen, and kidney) and tumors were collected. To assess the biodistribution of HCu, Cu concentration was measured in major organs and tumors using ICP-OES, after melting the tissue with chloroazotic acid.

### In vivo anticancer efficacy

The 4T1 breast cancer-bearing mice were randomly divided into seven groups (*n* = 5 in each group), and they are treated with intravenously (*i.v.*) administration of saline, intragastric (*i.g.*) administration of DSF plus copper gluconate (CuGlu) (*i.g.* DSF + CuGlu), DSF@HMON (*i.v.*), DSF@HCu (*i.v.*), PTT, HCu (*i.v.*) + PTT, and DSF@HCu (*i.v.*) + PTT (50 mg DSF/kg, 0.15 mg Cu/kg), respectively. The body weight and tumor volume were measured every other day after treatment.

## Supplementary Information

**Additional file 1: Methods. **1.1 In vitro DSF release of DSF@HMONs; 1.2 In vitro photothermal effect of CuS; 1.3 In vitro cytotoxicity assay of HMONs. **Figure S1.** The releasing profiles of DSF from DSF@HMONs in aqueous solutions. **Figure S2.** In vitro photothermal-conversion assessment of CuS. **Figure S3.** Relative cell viability of 4T1 cancer cells after incubation with different concentrations (0, 6.25, 12.5, 25, 50, 100, and 200 μg/mL) of HMONs for 24 and 48 h. **Figure S4.** Blood routine examination and serum biochemical levels of mice treated with 0 mg/kg, 5 mg/kg, 10 mg/kg, and 20 mg/kg of DSF@HCu. **Figure S5.** Histopathological examinations of major organs (heart, liver, spleen, lung, and kidney) from mice after different treatments.

## Data Availability

The data are available in the main manuscript, supplementary Information files, and from the corresponding authors upon reasonable request.

## Abbreviations

HMONs: Hollow mesoporous organosilica nanoparticles
DSF: Disulfiram
NIR: Near-infrared
DTC: Diethyldithiocarbamate
EPR: Enhanced permeability and retention
FDA: Food and Drug Administration
PTT: Photothermal therapy
TEOS: Tetraethyl orthosilicate
TEA: Triethanolamine
HCl: Hydrochloric acid
CTAC: Cetyltrimethylammonium chloride
MPTMS: 3-Mercaptopropyltrimethoxysilane
DPDS: 2, 2′-Dipyridyl disulfide
BTES: Bis(3-triethoxysilylproyl)disulfide
PI: Propidium iodide
CCK-8: Cell counting kit-8
FBS: Fetal bovine serum
H&E: Hematoxylin–eosin
HRP: Horseradish peroxidase
TEM: Transmission electron microscope
DLS: Dynamic light scattering
CLSM: Confocal laser-scanning microscope
ICP-OES: Inductively-coupled plasma optical emission spectrometer
FCM: Flow cytometry
CER I: Cytoplasmic extraction reagent I
CER II: Cytoplasmic extraction reagent II
NER: Nuclear extraction reagent
SDS-PAGE: Sodium dodecyl sulfate–polyacrylamide gel electrophoresis
PVDF: Polyvinylidene fluoride
