# Correction to: Co-delivery of nanoparticle and molecular drug by hollow mesoporous organosilica for tumor-activated and photothermal-augmented chemotherapy of breast cancer

**DOI:** 10.1186/s12951-021-01089-8

**Published:** 2021-11-03

**Authors:** Haixian Zhang, Feifei Song, Caihong Dong, Luodan Yu, Cai Chang, Yu Chen

**Affiliations:** 1 Department of Ultrasound, Department of Oncology, Fudan University Shanghai Cancer Center, Shanghai Medical College, Fudan University, 200032 Shanghai, People’s Republic of China; 2 Department of Pathology, Shanghai Tenth People’s Hospital Affliated To Tongji University, 200072 Shanghai, People’s Republic of China; 3 Department of Ultrasound, Zhongshan Hospital, Fudan University and Shanghai Institute of Medical Imaging, Shanghai, 200032 People’s Republic of China; 4 Materdicine Lab, School of Life Sciences, Shanghai University, Shanghai, 200444 People’s Republic of China

## Correction to: J Nanobiotechnol (2021) 19:290 https://doi.org/10.1186/s12951-021-01025-w

Following publication of the original article [1], the authors identified an error in the supplementary information. The correct version of supplementary information is given in this erratum.

**1. Supplementary methods**

**1.1 In vitro DSF release of DSF@HMONs**

DSF@HMONs (5 mg) was encapsulated into a dialysis bag (molecular weight cut-off = 5 kDa), and then immersed in aqueous solution (30 mL). The release procedure was conducted in a shaking table (100 rpm, 37 °C) and the DSF-releasing amount was measured by UV–vis spectroscopy at λ = 480 nm at the given time point.

**1.2 In vitro photothermal effect of CuS**

The photothermal performance of CuS was characterize by recording the temperature changes during laser irradiation with 808 nm (FLIR TM A325SC camera). CuS nanoparticles were dispersed in deionized water with different Cu concentrations (12.5, 25, 50, and 100 μg/mL), which were then exposed to 808 nm laser irradiation at the laser-power density of 1.5 W/cm^2^. In addition, the temperature increase of CuS aqueous solution at the Cu concentration of 50 μg/mL as irradiated by 808 nm laser at different power intensities (0.75, 1.0, 1.25, and 1.5 W/cm^2^) was tested.

**1.3 In vitro cytotoxicity assay of HMONs**

To evaluate in vitro cytotoxicity of HMONs, a standard CCK-8 viability assay was conducted. Varied concentrations (0, 6.25, 12.5, 25, 50, 100 and 200 μg/mL) of HMONs were co-incubated with 4T1 cells pre-seeded in 96-well plants for 6 h and 24 h. Then CCK-8 diluted by DMEM at a ratio of 1:10 was added into plates to test the cell viabilities at a wavelength of 450 nm after 60 min on a microplate reader.

**2. Supplementary figures**


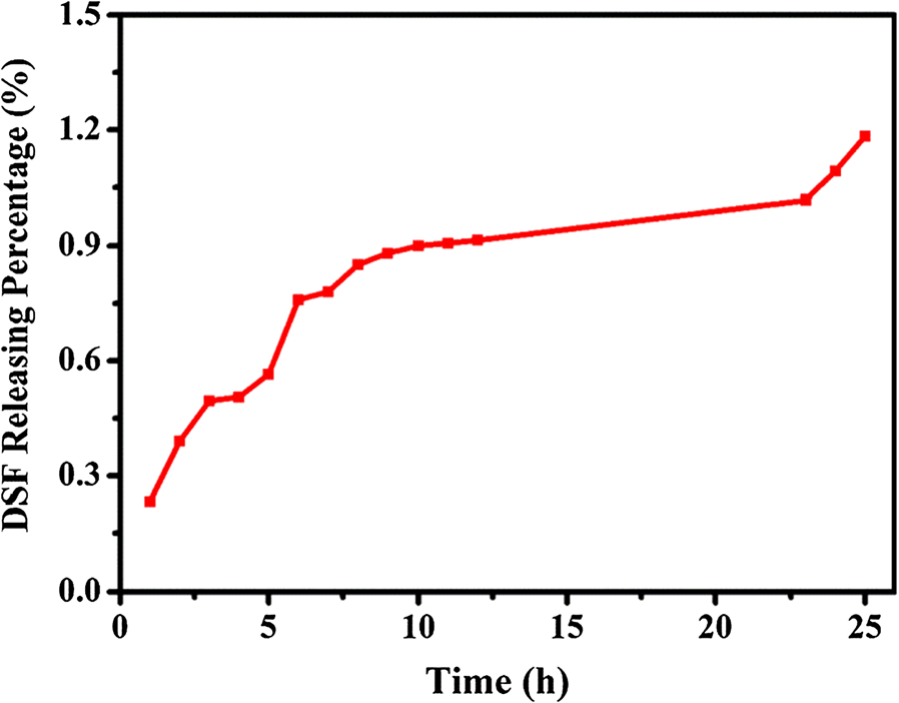


**Figure S1.** The releasing profile of DSF from DSF@HMONs in aqueous solution


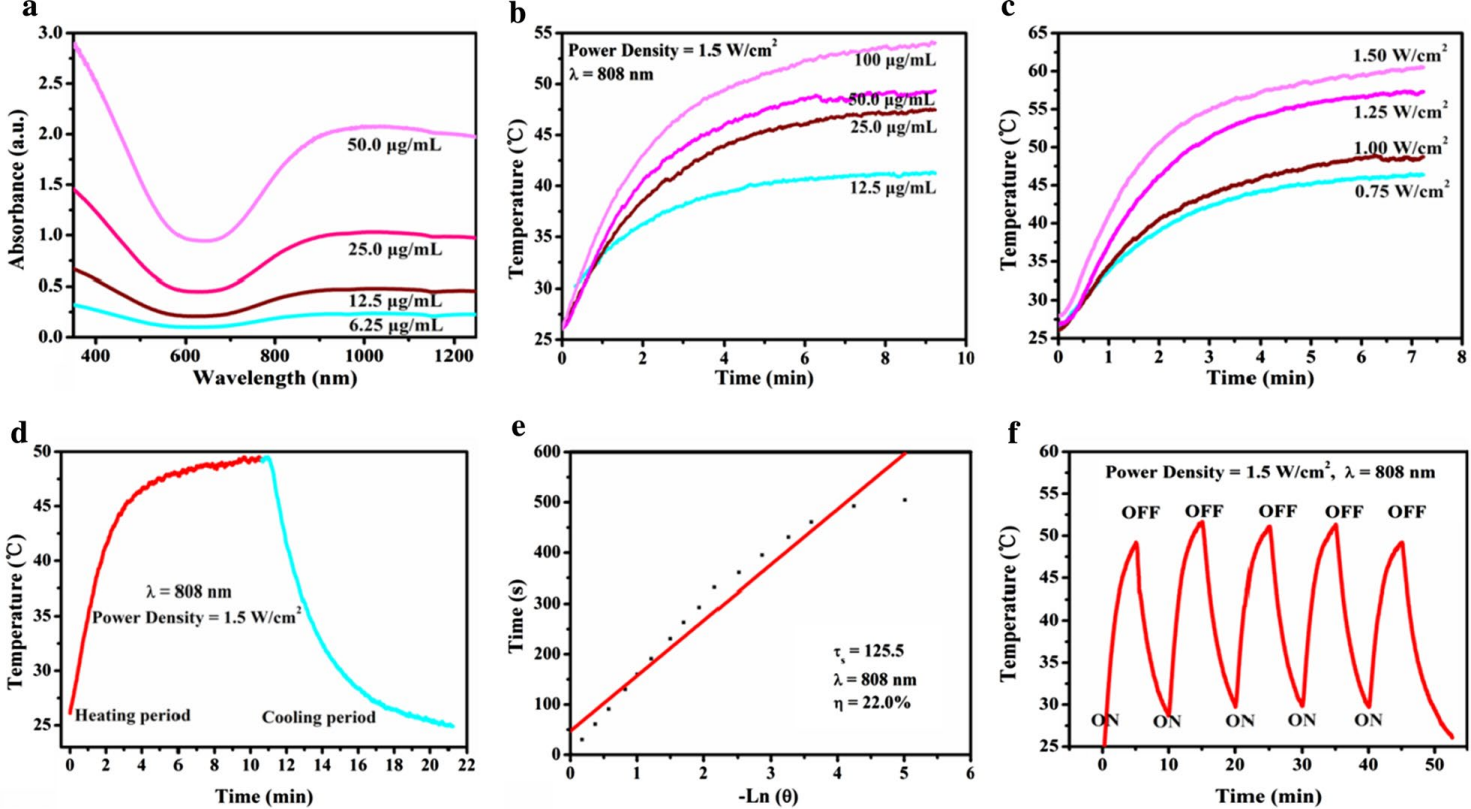


**Figure S2.** In vitro photothermal-conversion assessment of CuS. (a) UV–vis spectra of CuS at different concentrations (6.25, 12.5, 25 and 50 μg/mL) in aqueous solution. (b) Temperature changes of CuS aqueous solution with NIR laser (808 nm, power density: 1.5 W/cm^2^) irradiation at elevated concentrations (12.5, 25, 50, and 100 μg/mL). (c) Photothermal-heating curves of CuS dispersed in aqueous solution irradiated by different power intensities (0.75, 1.0, 1.25 and 1.5 W/cm^2^) of NIR laser at the wavelength of 808 nm. (d) Photothermal performance of CuS dispersed in aqueous solution under NIR irradiation; the laser was turned off when the temperature was stable. (e) Time constant for heat transfer calculated from the cooling period. (f) Heating curve of CuS dispersed in water for five laser on/off cycles irradiated by 808 nm laser at the power intensity of 1.5 W/cm^2^


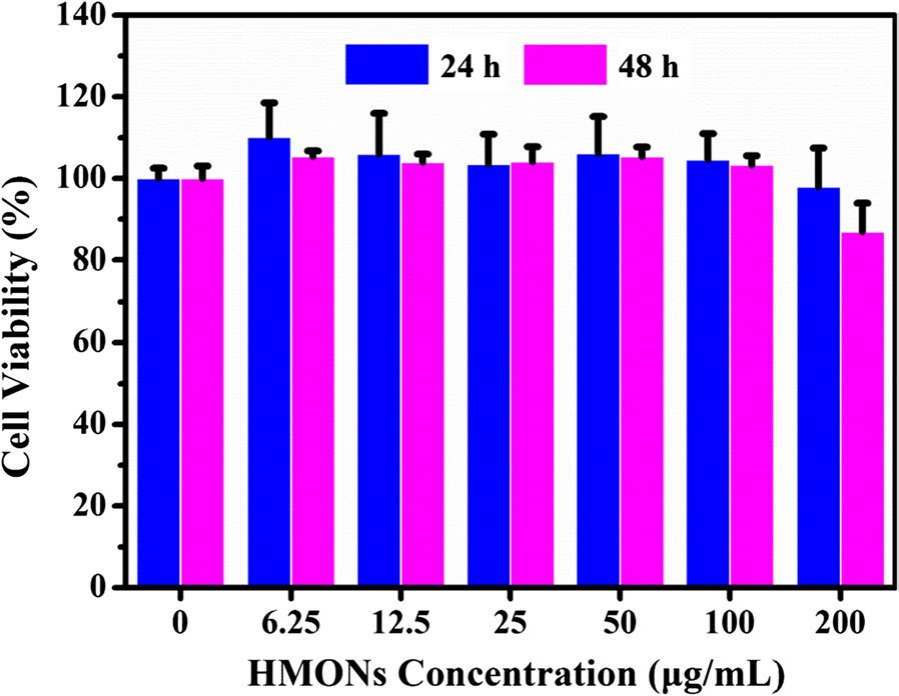


**Figure S3.** Relative cell viability of 4T1 cancer cells after incubation with different concentrations (0, 6.25, 12.5, 25, 50, 100 and 200 μg/mL) of HMONs for 24 h and 48 h


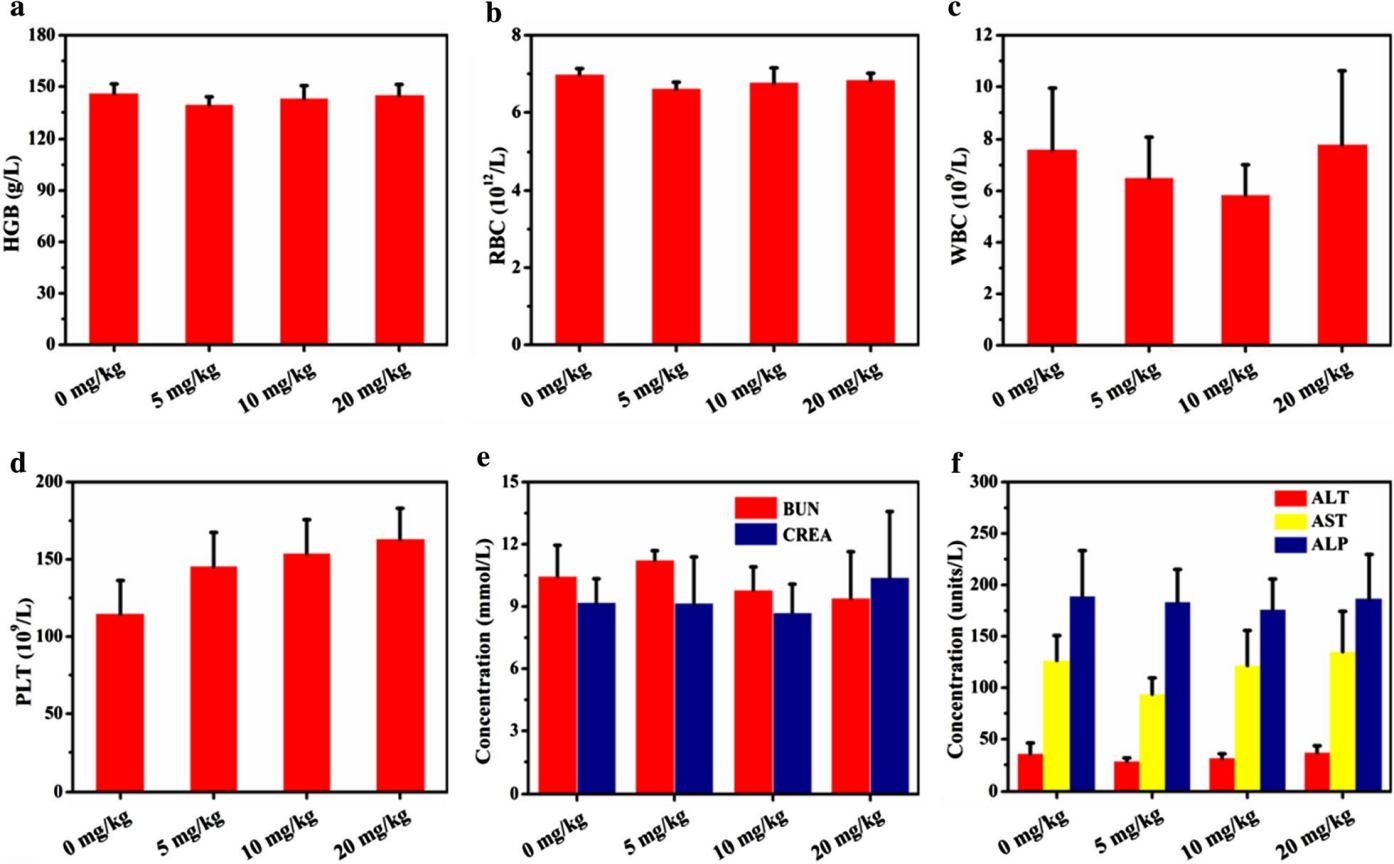


**Figure S4.** Blood routine examination and serum biochemical levels of mice treated with 0 mg/kg, 5 mg/kg, 10 mg/kg, and 20 mg/kg of DSF@HCu. (a) Hemoglobin (HGB), (b) Red blood cell (RBC), (c) White blood cell (WBC), (d) Platelet (PLT), (e) Blood urea nitrogen (BUN) and creatinine (CREA), (i) Alanine transaminase (ALT), aspartate aminotransferase (AST) and alkaline phosphatase (ALP) levels of mice in all groups


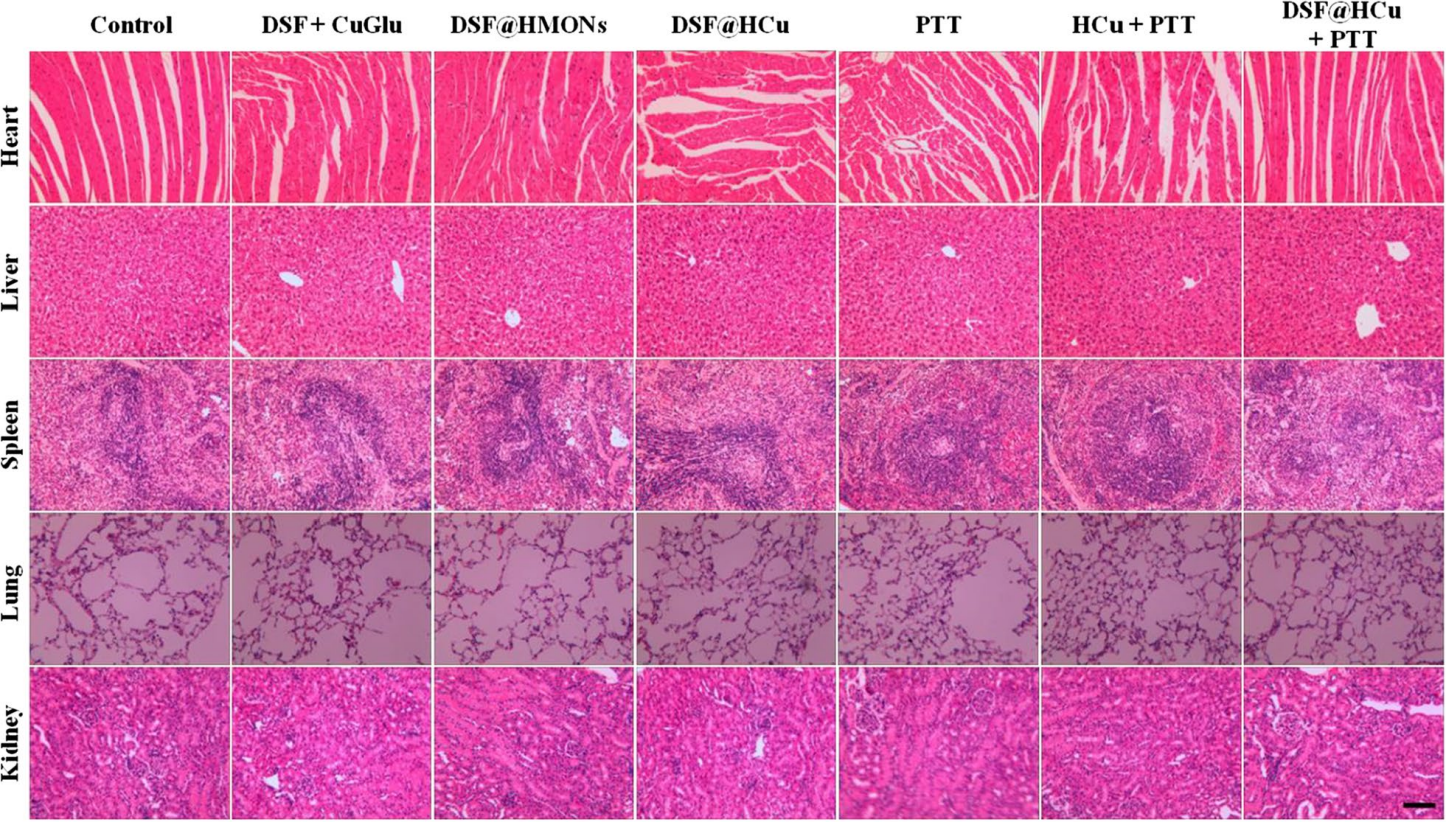


**Figure S5.** Histopathological examinations of major organs (heart, liver, spleen, lung and kidney) from mice after different treatments

The original article has been revised.
